# Gene Regulatory Network for Tapetum Development in *Arabidopsis thaliana*

**DOI:** 10.3389/fpls.2017.01559

**Published:** 2017-09-12

**Authors:** Dan-Dan Li, Jing-Shi Xue, Jun Zhu, Zhong-Nan Yang

**Affiliations:** College of Life and Environment Sciences, Shanghai Normal University Shanghai, China

**Keywords:** anther, tapetum, *DYT1*, *TDF1*, *AMS*, *MS188*

## Abstract

In flowering plants, male gametophyte development occurs in the anther. Tapetum, the innermost of the four anther somatic layers, surrounds the developing reproductive cells to provide materials for pollen development. A genetic pathway of *DYT1*-*TDF1*-*AMS*-*MS188* in regulating tapetum development has been proven. Here we used laser microdissection and pressure catapulting to capture and analyze the transcriptome data for the *Arabidopsis* tapetum at two stages. With a comprehensive analysis by the microarray data of *dyt1*, *tdf1*, *ams*, and *ms188* mutants, we identified possible downstream genes for each transcription factor. These transcription factors regulate many biological processes in addition to activating the expression of the other transcription factor. Briefly, *DYT1* may also regulate early tapetum development via E3 ubiquitin ligases and many other transcription factors. *TDF1* is likely involved in redox and cell degradation. *AMS* probably regulates lipid transfer proteins, which are involved in pollen wall formation, and other E3 ubiquitin ligases, functioning in degradating proteins produced in previous processes. *MS188* is responsible for most cell wall-related genes, functioning both in tapetum cell wall degradation and pollen wall formation. These results propose a more complex gene regulatory network for tapetum development and function.

## Introduction

Anther contains four locules, each with four layers of somatic cells surrounding the germ cells (Goldberg et al., 1993). The tapetum directly contacts the germ cells. It is the main tissue providing precursors for pollen development and pollen wall formation (Heslop-Harrison, 1962; Mariani et al., 1990; Ariizumi and Toriyama, 2011). Development of the tapetum is highly regulated. Tapetum cells are identified at anther stage 5 and undergo programmed cell death (PCD) in stage 10, then release the contents for further pollen wall development (Sanders et al., 1999). Mature pollen forms at stage 12 (Owen and Makaroff, 1995).

Several transcription factors regulating tapetum development and pollen formation have been reported. *DYSFUNCTIONAL TAPETUM1* (*DYT1*) encodes a putative basic helix-loop-helix (bHLH) transcription factor. Defective *DYT1* results in a premature vacuolation of the tapetum and the absence of pollen (Zhang et al., 2006). *DEFECTIVE in TAPETAL DEVELOPMENT and FUNCTION1* (*TDF1*) encodes a putative R2R3 MYB transcription factor. Defective *TDF1* results in a dysfunctional tapetum with irregular cell division and absence of pollen in both *Arabidopsis* and rice (Zhu et al., 2008; Cai et al., 2015). The transcription factor *ABORTED MICROSPORES* (*AMS*) encodes a bHLH family protein. The *ams* mutant shows degeneration of both tapetum and microspores at anther stage 7 (Sorensen et al., 2003; Xu et al., 2014). *AtMYB103*/*MS188*, also a member of the R2R3 MYB gene family, plays an important role in tapetum development, callose dissolution and exine formation in anthers. In *myb103*/*ms188* mutant, the tapetal protoplast contains several irregular enlarged vacuoles after meiosis stage, indicating the abnormal tapetal development in this mutant (Higginson et al., 2003; Zhang et al., 2007; Zhu et al., 2010, 2011).

Although the results of *in situ* hybridization with wild-type and mutant anther sections showed these transcription factors expressed in tapetum and microsporocytes, the GFP fusion proteins were specifically localized in tapetal cells (Gu et al., 2014; Xiong et al., 2016; Ferguson et al., 2017). The mutants of these four transcription factors are male-sterile, with defective tapetum development.

*DYT1* was expressed at anther stage 4, which indicates that it functions during the early stages of tapetum development (Zhu et al., 2011). Previous transcriptome analysis showed that *DYT1* integrates lipid metabolism, transporter, secondary metabolism and other biological processes for pollen development (Feng et al., 2012). *DYT1* directly regulates the expression of *TDF1* (Gu et al., 2014). The expression of *TDF1* in *dyt1* restored the expression of *AMS, MS188* and pollen wall-related genes although the transgenic line is still male sterile (Gu et al., 2014). *TDF1* was also expressed at stage 4 and peaked in expression in the tapetum and meiocytes at stage 6, then gradually decreased after meiosis. In *TDF1* knock out mutant, *AMS* is only minimally expressed, which indicates that *TDF1* acts upstream of *AMS* (Zhu et al., 2008, 2011). *AMS* is a key regulator of pollen wall formation. It directly regulates *MS188* and *transposable element silencing via AT-hook* (*TEK*) for sexine and nexine formation (Zhang et al., 2007; Lou et al., 2014). *AMS* expression was low at stage 5 but high at the meiosis stage and lasted until the release of pollen (Sorensen et al., 2003; Zhu et al., 2011). The expression pattern of *AMS* indicates that *AMS* might be responsible for both tapetum development at an early stage and degradation at a later stage. *MS188* is a key regulator of tapetum development and pollen sexine formation. In *ms188*, the degradation of the tapetum cell wall is delayed, and the sexine of the pollen wall is absent. Therefore, *MS188* is important for tapetum cell wall degradation and sexine formation (Zhang et al., 2007). These transcription factors form a genetic pathway, *DYT1*-*TDF1*-*AMS*-*MS188*, to regulate tapetum development and function (Zhu et al., 2008, 2011; Gu et al., 2014; Lou et al., 2014). However, the expression of *TDF1* in *dyt1* mutant cannot rescue their male-sterility, indicating the complexity of gene regulation network in tapetum development and pollen formation.

To better understand transcriptional regulation in the tapetum, we used laser microdissection and pressure catapulting (LMPC) to study gene expression in tapetum cells (Burgemeister et al., 2003; Zhan et al., 2015). With a comprehensive analysis of global transcriptional changes in *dyt1*, *tdf1*, *ams*, *ms188* mutants and transcriptomes of tapetum cells, we identified several subsets of genes as targets of the transcription factors in tapetum cells. Detailed analysis of this subset revealed an expanded transcriptional regulatory network during tapetum development in *Arabidopsis thaliana*.

## Materials and Methods

### Plant Material

Plants were grown under long-day conditions (16-h light/8-h dark) in a growth room at 22°C. The *dyt1-2*, *tdf1*, and *ms188* mutants were isolated from EMS mutation lines in the Ler background, then backcrossed to Col-0 more than 10 times, as described by Zhang et al. (2007), Zhu et al. (2008) and Song et al. (2009) and the *ams* mutant (SALK_152147) was obtained from the Arabidopsis Biological Resource Center (Ohio State University, Columbus, OH, United States) (Zhang et al., 2007; Zhu et al., 2008; Song et al., 2009).

### Tissue Preparation

Farmer‘s fixative (Kerk et al., 2003) [75% (v/v) ethanol and 25% (v/v) acetic acid] was freshly prepared and chilled in a -20°C freezer for at least 20 min. In total, 20∼30 inflorescences of 6- to 8-week-old *A. thaliana* ecotype Columbia-0 were immediately immersed in the fixative solutions in a glass vial and fixed under a soft vacuum for 15 min on ice three times with fresh fixative.

Paraffin-embedding was followed by the microwave method reported by Inada and Wildermuth (2005) with minor modification. The fixative solution in the vial was replaced with a new pre-chilled fixative solution before the microwave method. The tissues was microwaved in the fixative solution at 450 W at 37°C for 15 min three times, each time replaced with a new fixative solution. The fixed samples were dehydrated with 75% ethanol at 67°C for 1 min 15 s, followed by dehydration steps of 1 min 15 s each in 85, 95, 100, and 100% ethanol. Several drops of 1.0% Safranin-O were added to the second 100% ethanol step (Ruzin, 1999). The 100% ethanol was replaced with ethanol: xylene 1:1 followed by 100% xylene with each step microwaved at 70°C for 1 min 30 s. Xylene was subsequently replaced with paraplast, and samples in xylene : paraplast 1:1 were microwaved at 70°C for 10 min. Then, samples were microwaved in 100% paraplast at 70°C for 10 min, then at 70°C for 2.5 h, with paraplast replaced every 30 min. The samples were placed into a Kraft dish filled with 100% new paraplast, and embedded samples were cooled to room temperature. After cooling, the paraplast blocks were stored at 4°C.

### Laser Microdissection and Pressure Catapulting (LMPC)

We obtained 8-μm-thick sections by using a rotary microtome (Leica RM2235; Leica Bio-systems Nussloch). To enhance RNA preservation, a paraffin tape transfer system (Instrumedics) was used (Cai and Lashbrook, 2006). Before LMPC, the slides were illuminated for 1 min under an ultraviolent lamp (366 nm) in the paraffin tape transfer system and deparafinized twice in xylene, 10 min each time, and air dried for 2 to 3 h.

Laser microdissection and pressure catapulting involved use of a PALM MicroLaser system (PALM-Zeiss, Bernried, Germany) containing a PALM MicroBeam (driven by PALM MicroBeam software) and a PALM RoboStage and, for high-throughput sample collection, a PALM RoboMover (driven by PALM RoboSoftware v4.6). The slides were then loaded into the PALM Microbeam (Carl Zeiss, Germany). The tapetum cells of *Arabidopsis* anther on the slides were first selected by using the graphic tools of the PALM RoboSoftware. Then cells were collected by laser pressure, which catapults them into the cap of an AdhesiveCap (Carl Zeiss, Germany). The tapetum cells at stages 6 and 7 were placed into one cap and cells at stages 8, 9, and 10 in another cap. Approximately 1 mm^2^ (about 2000 cells) of tissue was obtained for each sample per library.

### RNA Extraction, Amplification, and RNA-Seq

For the two samples, total RNA was extracted by using a PicoPure RNA isolation kit (Acturus) and subjected to DNase treatment (RNase-free DNA set; Qiagen) according to the manufacturer’s protocol. Next, 10 ng RNA was used for cDNA synthesis and amplification by using an Ovation RNA-seq System V2 kit (NuGen Technologies) following the manufacturer’s protocols with minor modifications. The quality and profile of the RNA and amplified cDNA was assessed by using an RNA 6000 Pico Assay Kit with an Agilent 2100 Bioanalyzer, respectively (Agilent Technologies). The cDNA libraries were then further refined by using the Illumina TruSeq RNA Sample Preparation v2 kit (Illumina) with the low-throughput protocol according to the manufacturer’s instructions. Then samples were paired-end sequenced by using an Illumina HiSeq 2500 platform (Illumina Inc.)^1^. The average length of the reads was 125 bp.

### Read Mapping and Analysis

RNA-seq reads were mapped to the *Arabidopsis* genome (TAIR10^2^) by using TOPHAT v2.0.9 (Trapnell et al., 2009). Raw reads were preprocessed to remove adaptors using an in-house developed Perl script. After filtering out low-quality (lowest base score < 30) reads using SolexaQA v3.1.3 (Cock et al., 2010). The multi-mapped read correction and fragment bias correction options of Cufflinks v2.1.1 (Trapnell et al., 2010) were used. Gene expression levels were reported as fragments per kilobase of transcripts per million mapped reads [FPKM; (Mortazavi et al., 2008)]. The upper- and lower-bound FPKM values (FPKM_conf_hi and FPKM_conf_lo, respectively) for the 95% confidence interval of each gene were provided by Cufflinks. A gene was defined as expressed in a sample if the FPKM_conf_lo was >0. Differential gene expression between stages 6–7 and stages 8–10 was evaluated using the R package edgeR.

### Microarray Analysis

Closed floral buds were collected from wild-type and *dyt1, tdf1, ams* mutant plants for RNA isolation. Three separate microarrays were performed for each mutant. Total RNA was isolated by use of TRIzol reagent (Invitrogen), and further purified by using the RNeasy Mini kit (Qiagen). Three biological replicates of total RNA were used. Total RNA was labeled by Cy3 and Cy5 with use of the Low RNA Input Linear Amplification and Labeling kit plus (Agilent, 5184-3523). After cRNA labeling, samples were hybridized onto a 44 K *Arabidopsis* oligo microarray by using the Gene Expression Hybridization kit (Agilent, 5188-5242; Agilent, Santa Clara, CA, United States). Arrays were scanned by using the Agilent G2565BA laser scanner. The microarray data for the *ms188* mutant were obtained from a previous study (Zhu et al., 2010). Significance analysis of microarrays (SAM) was used to calculated gene expression values and differentially expressed genes. In this study, the threshold value were fold change > 2, FDR < 0.05, and *q*-value < 0.05. The delta cut-off of *dyt1* was 1.43, while that of *tdf1*, *ams, ms188* was 2.2, 1.55, and 1.47, respectively.

### Functional Classification and Categorization of Expression Patterns

MAPMAN analysis was performed as described (Thimm et al., 2004). Then hypergeometric distribution was used to calculate the p value for Gene Ontology (GO) enrichment analyses. Cluster analysis involved use of MEGA^3^. Heatmap analysis involved use of R^4^.

### Real-time PCR

Real-time PCR analysis was performed as described (Lou et al., 2014). Three biological replicates for each sample and three technical replicates for each gene were used. The relevant primers are in Supplementary Table S19. The β-tubulin gene was used as a positive control.

## Results

### RNA Sequencing with LMPC Captured Tapetum Cells at Anther Stages 6–7 and 8–10

**FIGURE 1:**
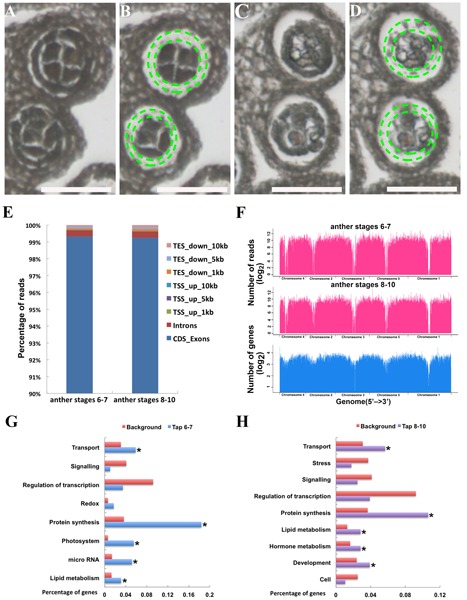
RNA sequencing and Gene Ontology (GO) enrichment analysis of transcriptome from laser microdissection and pressure catapulting (LMPC) captured tapetal cells of anther at stages 6–7 and 8–10. **(A)** Wild-type Col-0 anther at stages 6–7 before LMPC. **(B)** Wild-type Col-0 anther at stages 6–7 after LMPC, tapetal cells were isolated. **(C)** Wild-type Col-0 anther at stages 8–10 before LMPC. **(D)** Wild-type Col-0 anther at stages 8–10 after LMPC, tapetal cells were isolated. **(E)** Reads distribution in different gene area. Over 99% of the reads were mapped in the CDS_Exons. TES, transcription termination site; TSS, Transcription start site; CDS, coding DNA sequence. **(F)** Reads distribution in the whole genome, compared with the gene distribution in the whole genome. **(G)** GO enrichment analysis of tapetum expressed genes at anther stages 6–7. **(H)** GO enrichment analysis of tapetum expressed genes at anther stages 8–10. Asterisk indicates *P* values < 0.05 compared with background.

Tapetum in *A. thaliana* was identified at anther stage 5 and initiated degradation at stage 10 (Sanders et al., 1999). To identify genes expressed in both early- and later-stage tapetum cells, we used LMPC to capture tapetum cells at anther stages 6–7 and 8–10, respectively (**Figures 1A–D**). RNAs of the captured tapetum cells were reverse-transcribed to cDNA, then used to make Illumina RNA-seq libraries and sequenced. Over 99% reads could be mapped to the *Arabidopsis* reference CDS database (99.3% at anther stages 6–7, total reads 53 million; and 99.2% at anther stages 8–10, total reads 50 millions), covered most of the *Arabidopsis* reference genome (**Figures 1E,F**). The mapped reads were normalized by using fragments per kilobase per million reads (FPKM) (Trapnell et al., 2010). We used FPKM 95% confidence interval > 0 as the standard for an expressed gene (Zhan et al., 2015). With this criterion, 18,298 genes were expressed in the tapetum at anther stages 6–7 and 18,227 at anther stages 8–10 (Supplementary Data S1, S2). Of these genes, 15,977 (77.8%) genes were expressed both at early and later stages. Most of the known tapetum expressed genes were confirmed in the sequencing data (Supplementary Table S1). With a threshold of 300 FPKM as extremely high-expressing genes, 292 genes were found in the tapetum at anther stages 6–7 and 283 at anther stages 8–10 (Supplementary Data S3, S4). Among those genes, 187 (48.2%) genes were highly expressed both at the two stages. We used GO and MapMan analysis for these genes (Thimm et al., 2004; Usadel et al., 2005). Protein synthesis, transporters, and lipid metabolism genes were highly expressed in the tapetum at both stages 6–7 and 8–10, which indicates a highly dynamic metabolism and transportation system of tapetum for supporting pollen development (Piffanelli et al., 1998; Ariizumi and Toriyama, 2011) (**Figures 1G,H** and Supplementary Tables S2, S3). At anther stages 8–10, the expression of pectinases, G-protein genes and 1-aminocyclopropane-1-carboxylate synthase 4 (*ACS4*) were highly upregulated. The primary plant cell wall is a complex mixture of polysaccharides and proteins encasing living plant cells. The pectinases act as glycoside hydrolases for plant cell wall degradation (Cosgrove, 2005; Kirsch et al., 2012). After meiosis, the tapetal cells become a spongy shape with cell wall degradation in wild-type anthers (Zhang et al., 2007; Zhu et al., 2010). The accumulation of pectinases in tapetal cell is consistent with this cytological character. The GTP-binding proteins (G proteins) are conserved signal transduction molecules as cytokinin signaling in animals and plants. In *Arabidopsis*, mutation in three of extra-large G proteins (XLGs) leads to an abnormally expanded tapetum phenotype, indicating the G proteins were involved in tapetal development (Wang et al., 2017).

### Identification of Tapetum-Expressed Downstream Genes of *DYT1*, *TDF1*, *AMS*, and *MS188*

**FIGURE 2:**
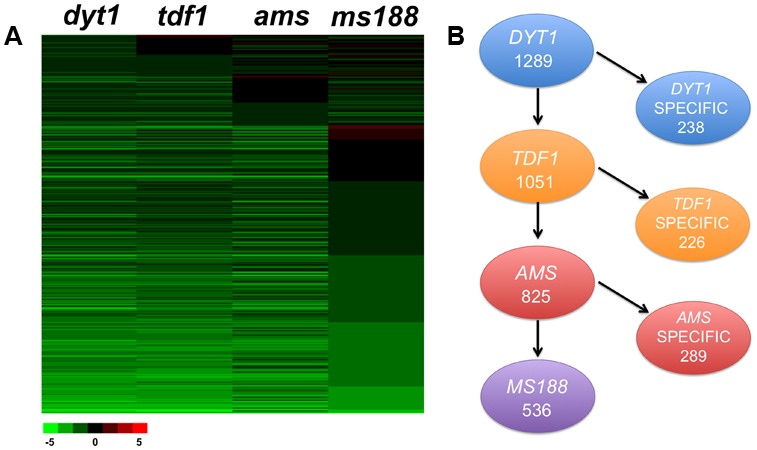
Comparative analysis of tapetum expressed genes differentially down-regulated in *dyt1, tdf1, ams*, and *ms188* mutants. **(A)** Transcription changes of tapetum expressed genes differentially down-regulated in *dyt1, tdf1, ams*, and *ms188* mutants. Green and red colors represent lower and higher log2 fold change expression in mutants compared with the wild type. **(B)** Subsets of total differentially and commonly expressed genes down-regulated in the four mutants.

Previous investigations showed that the transcription factors *DYT1*, *TDF1*, *AMS*, and *MS188* are key regulators in tapetum development (Sorensen et al., 2003; Zhang et al., 2006, 2007; Zhu et al., 2008). As these transcription factors act as the transcriptional activators, we mainly focus on the downregulated genes in current study (Feng et al., 2012; Lou et al., 2014; Xiong et al., 2016). Recently we obtained the transcriptional profiling data of flower buds in these mutants. Significance analysis of microarrays (SAM) (Tusher et al., 2001) was used to identify differentially expressed genes in mutants more than twofold change compared with wild-type. Totally, 2179 genes in *dyt1*, 1953 genes in *tdf1*, 1508 genes in *ams* and 1287 genes in *ms188* were identified to be down-regulated, respectively (FDR < 0.05 and *q*-value < 0.05). To further analyze the transcriptional regulation of tapetum development, we compared the expressed genes in tapetum transcriptome data (FPKM > 2) and the flower buds transcriptional profiling of *dyt1, tdf1, ams*, and *ms188* mutants (Zhang et al., 2006; Zhu et al., 2008; Feng et al., 2012) (**Figure 2A**). In total, 1289 significantly down-regulated genes in *dyt1* were expressed in the tapetum, including 1051 genes that were also down-regulated in *tdf1*, 825 genes that were affected in *dyt1*, *tdf1* and *ams*, and 536 genes that were altered in *dyt1*, *tdf1*, *ams*, and *ms188* (**Figure 2B** and Supplementary Data S5). We defined the genes expressed in tapetum and down-regulated in *dyt1* but not in *tdf1* as *DYT1*-specific regulated genes, genes down-regulated in both *dyt1* and *tdf1* but not in *ams* as *TDF1*-specific regulated genes. With such a definition, 238 *DYT1*, 226 *TDF1*, and 289 *AMS* specific genes were identified (**Figure 2B** and Supplementary Data S6–S8). 536 down-regulated genes in all four mutants were defined as common genes (**Figure 2B** and Supplementary Data S9). The transcriptomes of uninucleate microspores (UNM), bicellular pollen (BCP), immature tricellular pollen (TCP) and mature pollen grains (MPG) from Honys and Twell (2004) were analyzed in a recent study, comparing to the genes expressed in tapetal cells (Supplementary Data S10). We found thousands of microspores expressed genes were not expressed in tapetal cells. We used GO and MapMan to further analyze the *DYT1-*, *TDF1-*, *AMS-* specific and common genes. The subsets of these specific genes indicated that besides the activation of the downstream genes, each transcription factor might regulate several processes individually.

### Functional Analysis of *DYT1*-Specific Genes

**FIGURE 3:**
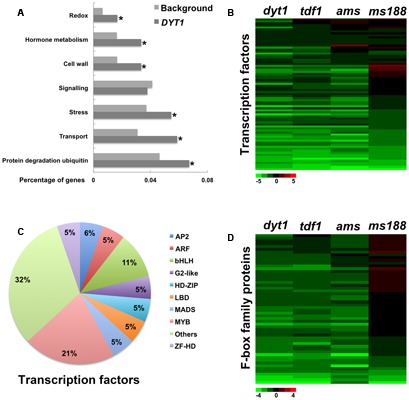
Functional analysis of *DYT1*-specific genes. **(A)** GO enrichment analysis of *DYT1*-specific genes. **(B)** Heat map of mRNA expression profiles of transcription factors of *DYT1* in *dyt1*, *tdf1*, *ams*, and *ms188* mutants. Green and red colors represent lower and higher log_2_ fold change of expression in mutants compared with the wild type. **(C)** Subsets in *DYT1*-specific transcription factors. **(D)** Heat map of mRNA expression profiles of F-box family proteins of *DYT1* in *dyt1*, *tdf1*, *ams*, and *ms188* mutants. Green and red colors represent lower and higher log2 fold change of expression in mutants compared with the wild type. Asterisk indicates *P* values < 0.05 compared with background.

Among the 1289 down-regulated genes in *dyt1*, 81.5% (1051) were also down-regulated in *tdf1*, including most of the lipid metabolism related genes, transporters, and cell wall related genes, indicating *TDF1* is the main target of *DYT1*. The *DYT1*-specific genes included transcription factors, E3 ubiquitin ligases and transporters (**Figure 3A** and Supplementary Table S4). *DYT1* is the earliest known regulator of the tapetum cell (Zhu et al., 2011). Although *DYT1* regulates downstream genes primarily via *TDF1* (Gu et al., 2014), the enrichment of transcription factors in *DYT1*-specific genes indicates that *DYT1* might play other important functions for tapetum development independent of *TDF1* (**Figures 3B,C** and Supplementary Table S5). Among them, two bHLH genes, *bHLH089* and *bHLH010* have been identified as *DYT1*-specific genes. Previous study showed that *bHLH010*, *bHLH089*, and *bHLH091* were redundantly required for *Arabidopsis* anther development. *DYT1* was required to activate the expression of these genes (Zhu et al., 2015; Cui et al., 2016). The enrichment of E3 ubiquitin ligases in *DYT1-*specific genes suggested that they may degrade some proteins to maintain tapetum-cell identity (**Figure 3D** and Supplementary Table S6). Previous studies has been reported that the transport and signaling of auxin are essential for anther and pollen development (Yang et al., 2013; Cardarelli and Cecchetti, 2014). In the hormone group, auxin-responsive GH3 family gene GH3.3, small auxin upregulated RNA SAUR25 and SAUR30 were enriched (Supplementary Table S7). GH3 and SAUR gene family are primary auxin response gene family (Hagen and Guilfoyle, 2002). *DYT1* might affect auxin signal response in tapetum at early stages.

### Functional Analysis of *TDF1*-Specific Genes

**FIGURE 4:**
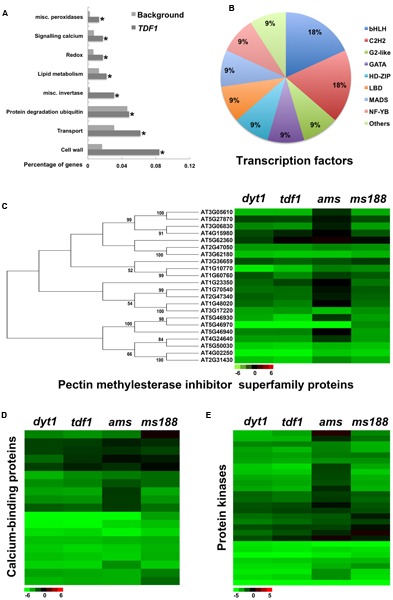
Functional analysis of *TDF1*-specific genes. **(A)** GO enrichment analysis of *TDF1*-specific genes. **(B)** Subsets of transcription factors in *TDF1*-specific genes. **(C)** Cluster and heat map of mRNA expression profiles of pectin methylesterase inhibitor superfamily proteins of *TDF1* in *dyt1*, *tdf1*, *ams*, and *ms188* mutants. **(D)** Heat map of mRNA expression profiles of calcium-binding proteins of *TDF1* in *dyt1*, *tdf1*, *ams*, and *ms188* mutants. Green and red colors represent lower and higher log_2_ fold change of expression in mutants compared with the wild type. **(E)** Heat map of mRNA expression profiles of protein kinases of *TDF1* in *dyt1*, *tdf1*, *ams* and *ms188* mutants. Asterisk indicates *P* values < 0.05 compared with background.

Among the 1051 genes down-regulated in the *dyt1* and *tdf1* mutants, 825 (78%) were also down-regulated in *ams*, including most of the lipid-metabolism and cell wall-related genes (Supplementary Data S5). The remaining 226 genes were termed *TDF1*-specific genes. Transcription factors, protein degradation related genes, redox proteins, cell wall-related genes and transporters were enriched in *TDF1*-specific genes (**Figure 4A** and Supplementary Table S8). 11 *TDF1*-specific transcription factors were identified (**Figure 4B** and Supplementary Table S5). Tapetum degradation requires temporal control of reactive oxygen species (ROS). NADPH oxidases *RESPIRATORYBURST OXIDASE HOMOLOG* (RBOH) genes and glutaredoxins (GRX) genes have been proved to regulate tapetum degradation (Xing and Zachgo, 2008; Xie et al., 2014). The tapetum degradation in *dyt1* and *tdf1* mutants is also abnormal. The vacuolated tapetum cells were enlarged and occupied the locular at anther stage 11 in *dyt1* and *tdf1* mutants, suggested the irregular PCD process of them (Zhang et al., 2006; Zhu et al., 2008, 2011). Plant glutathione-*S*-transferase (GST) proteins can protect tissues from oxidative damage (Marrs, 1996). Peroxidases are important for the redox regulation in plants (Apel and Hirt, 2004). Catalases has been reported to be an major type of ROS-scavenging enzymes (Mittler et al., 2004). All GSTs and catalases down-regulated in *dyt1* were also *TDF1*-specific genes (Supplementary Table S9). There were five peroxidases down-regulated in *dyt1*, and three of them were also *TDF1*-specific genes (**Supplementary Figure S1** and Table S9). Thus *TDF1* might functional in maintaining the redox balance in tapetum. The cysteine protease is involved in tapetum PCD and pollen development (Zhang et al., 2014). Metacaspases have been proposed to be involved in regulating PCD (Yang et al., 2014; Zhang et al., 2014; Fagundes et al., 2015; Daneva et al., 2016). We also found metacaspases and cysteine proteases enriched in *TDF1*-specific genes (Supplementary Table S9). These proteins may play a role in tapetum PCD. Several reports indicated that calcium-binding proteins contribute to tapetum development and degradation (Falasca et al., 2013; Wei et al., 2015; Monihan et al., 2016). Genes involved in calcium signaling were also enriched in *TDF1*-specific genes (**Figures 4D** and Supplementary Table S10). The leucine-rich repeat receptor-like kinases have been reported to be involved in anther somatic layer differentiation including tapetum in male gametogenesis (Zhao et al., 2002; Albrecht et al., 2005; Mizuno et al., 2007). Receptor kinases and protein kinases/phosphatases were enriched, so *TDF1* might regulate tapetum signaling communication (**Figures 4E** and Supplementary Table S11). Pectin methylesterases (PMEs) remove the methylesterase of pectin. PMEs are antagonistically regulated by PME inhibitors (PMEIs) (Levesque-Tremblay et al., 2015). Most of the down-regulated PMEIs in *dyt1* mutant were *TDF1*-specific genes (**Figure 4C**), indicate that *TDF1* might also regulate the pectin methylesterase in tapetum cell wall.

### Functional Analysis of *AMS*-Specific Genes

**FIGURE 5:**
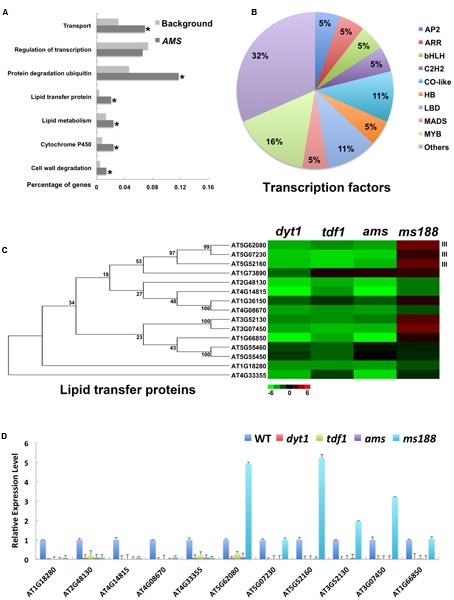
Functional analysis of *AMS*-specific genes. **(A)** GO enrichment analysis of *AMS*-specific genes. **(B)** Subsets of transcription factors in *AMS*-specific genes. **(C)** Heat map of mRNA expression profiles of lipid transfer proteins of *AMS* in *dyt1*, *tdf1*, *ams*, and *ms188* mutants. Green and red colors represent lower and higher log_2_ fold changes of expression in mutants compared with the wild type. **(D)** Q-RT-PCR analysis of the lipid transfer proteins. Asterisk indicates *P* values < 0.05 compared with background.

Among the 825 genes downregulated in the tapetum of *dyt1*, *tdf1*, and *ams* mutants, 65% (536) were also downregulated in *ms188*. Therefore, *MS188* is the major regulator under *AMS*. However, the expression of *MS188* in *ams* cannot rescue its male-sterility phenotype (Xiong et al., 2016). Therefore, *AMS*-specific genes are also important for tapetum development. *AMS*-specific genes contain few cell-wall, hormone-metabolism and transcription-factor genes (**Figures 5A,B** and Supplementary Table S12). E3 ubiquitin ligases were highly enriched (**Figure 3D**). Among all the 53 F-box E3 ligases in *dyt1*, 43% were *AMS*-specific genes. These E3 ligases might function in tapetum degradation. Thus the expression of *AMS* might transfer the tapetum cell fate into cell death by terminating several key factors via protein degradation. Type III lipid transfer proteins (LTPs) are expressed in the tapetum and function in pollen exine formation (Huang et al., 2013). Lipid metabolism-related genes were enriched in *AMS*-specific genes. We found 15 genes coding for LTPs were down-regulated in *dyt1*. Six of them were *AMS*-specific genes, including the three type III LTPs. Some other LTPs were *MS188*-specific genes (**Figure 5C** and Supplementary Table S13). Further qRT-PCR analysis confirmed these results (**Figure 5D**). The expression of LTPs might be mainly regulated by *AMS* and *MS188*. As well, lipid transfer might start at the *AMS* stage, which might function in preparation for pollen wall formation.

### Functional Analysis of the Common Genes

**FIGURE 6:**
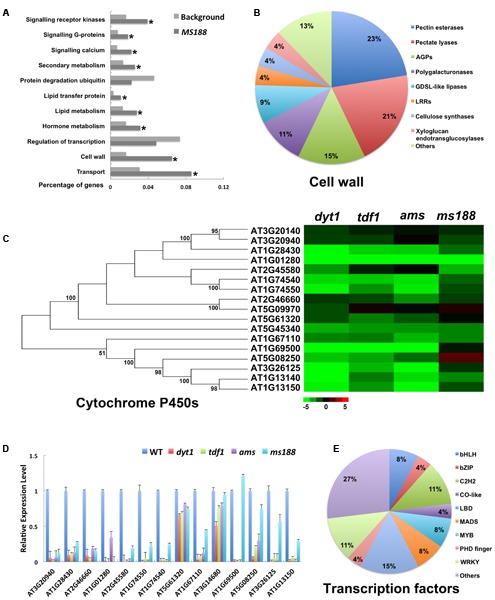
Functional analysis of commonly expressed genes down-regulated in the four mutants. **(A)** GO enrichment analysis of common genes. **(B)** Subsets of cell wall related genes in common genes. **(C)** Heat map of mRNA expression profiles of cytochrome P450 family genes in *dyt1*, *tdf1*, *ams*, and *ms188* mutants. **(D)** Q-RT-PCR analysis of the cytochrome P450 family genes. **(E)** Subsets of transcription factors in common genes. Asterisk indicates *P* values < 0.05 compared with background.

Among the 536 genes down-regulated in all the four mutants, cell-wall related genes were highly enriched and E3 ubiquitin ligases were reduced (**Figure 6A** and Supplementary Table S14). At late stage of anther development, tapetum cell wall is degraded, and *MS188* is important for this degradation (Zhang et al., 2007). Pectate lyases and polygalacturonases (PGs), and xyloglucan endo-transglucosylases are typical cell wall hydrolytic enzymes (Carpita and McCann, 2000; Kim et al., 2006). These proteins contributed to 36% of all cell wall related genes (**Figure 6B** and Supplementary Table S15). They may be responsible for tapetum cell wall degradation. Arabinogalactan proteins (AGPs), and fasciclin-like AGPs (FLAs) function in pollen wall formation (Li H. et al., 2010; Li J. et al., 2010; MacMillan et al., 2010; Tan et al., 2013; Jia et al., 2015; Showalter and Basu, 2016). We found AGPs and FLAs enriched in common cell wall genes (**Figure 6B**). AGPs are essential for *TEK*-mediated pollen wall formation (Jia et al., 2015). *TEK* is highly expressed in the tapetum at stage 7 and greatly reduced at stage 8 (Lou et al., 2014). AGPs were also down-regulated in *ms188*, so AGPs might contribute to the pollen wall formation after stage 7, and *MS188* might regulate the transcription of AGPs at later stages. Cytochrome P450 is a superfamily of heme-containing proteins that catalyze NADPH and O_2_-dependent monooxygenase (Chapple, 1998; Coon, 2005). Several cytochrome P450s were reported to contribute to the development of pollen wall (Morant and Bak, 2007; Dobritsa et al., 2009; Li H. et al., 2010; Amrad et al., 2016; Xiong et al., 2016). Here we found 17 cytochrome P450s down-regulated in *dyt1* in the tapetum, and most of them were common genes (**Figure 6C** and Supplementary Table S16). *MS188* functions in both degradation of the tapetum cell wall and formation of the pollen wall (Zhou et al., 2015; Xu et al., 2016). Further qRT-PCR analysis confirmed these results (**Figure 6D**). Many transcription factors, especially zinc finger and LOB domain-containing protein (LBD) family transcription factors were common genes (**Figure 6E** and Supplementary Table S5). The receptor kinases, calcium-related proteins, and G-proteins were still enriched in common genes. Rapid alkalization factor (RALF) proteins are peptides that function to regulate plant development (Sharma et al., 2016). We also found RALF proteins enriched in *MS188*-specific genes (**Supplementary Figure S2**). The tapetum might be both a target and a source of peptide signaling at the *MS188* stage.

## Discussion

**FIGURE 7:**
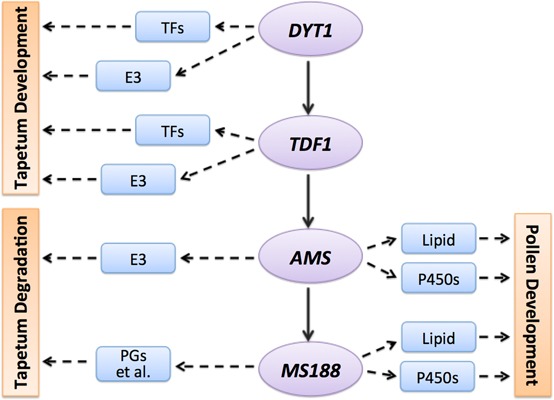
A working model of *DYT1-TDF1-AMS-MS188* pathway. Combined of our analysis, here we hypothesis a simple model of how different transcription factor regulate the tapetum and pollen development. TFs, transcription factors; E3, E3 ubiquitin ligases; P450s, cytochrome P450 genes; PGs, polygalacturonases.

During anther development, tapetum provides nutrition for pollen development and materials for pollen wall formation. It only lasts for several days from its differentiation from inner secondary parietal cells to its completely degradation of PCD (Feng and Dickinson, 2010a). In *Arabidopsis*, *DYT1-TDF1-AMS-MS188* form a genetic pathway for tapetum development and pollen wall formation (Zhu et al., 2011). After laser microdissection and sequencing of tapetum cells, and integration of transcriptional profiling of *dyt1, tdf1, ams*, and *ms188* mutants, we identified reliable tapetum expressed genes, and specific genes regulated by these transcription factors (**Figure 7**). Although only a subset of the specific genes for each transcription factor are likely to be direct targets of the TFs, these informations provide novel understandings of the *DYT1*-*TDF1*-*AMS*-*MS188* pathway in a highly ordered and precisely regulated network for tapetum and pollen development.

In this work, we found that 536 tapetum genes were common downstream genes of all the four transcription factors, and each factor regulated hundreds of specific genes independent of the downstream components of the pathway. The down-regulated transcriptional data of tapetum genes were highly similar between *dyt1* and *tdf1* mutant (81.5%); *tdf1* and *ams* mutant (78%), *ams* and *ms188* mutant (65%). This indicates that these transcription factors play their functions mainly through downstream key regulators for tapetum development. However, each transcription factor should play some specific functions as the downstream genes could not rescue the pollen formation (Gu et al., 2014; Xiong et al., 2016). Besides the common regulated genes, there were 238 *DYT1*-specific genes, 226 *TDF1*-specific genes, 289 *AMS*-specific genes. The identification of these specific genes is helpful to understand the specific functions of these regulators. Of the 74 transcription factors down-regulated in *dyt1* mutant, 19 were *DYT1*-specific genes, 11 were *TDF1*-specific genes, and 19 were *AMS*-specific genes (Supplementary Table S5). This indicates that besides the primary targets, each factor might regulate other TFs for tapetum development. E3 ubiquitin ligase is widely reported to degrade proteins during plant development. In *Arabidopsis*, E3 ligase PUB4 was reported to involve in tapetal cell development (Wang et al., 2013). *DYT1*, *TDF1*, and *AMS* are early regulators for tapetum development. All these transcription factors regulate some specific E3 ligases (Supplementary Table S6). E3 protein-degradation system may degrade some of the former-stage proteins to maintain tapetum cell identity at a specific stage to ensure successful tapetum development. AMS is critical for tapetum development as the *ams* tapetum cell is enlarged to occupy the anther locule (Sorensen et al., 2003). Most of the E3 ligase were *AMS*-specific genes (**Figure 3D**). It’s likely that *AMS* plays the role in determining the tapetum cell fate via this E3 ligase protein degradation. Many receptor kinases are important regulators in *Arabidopsis* tapetum and pollen development (Zhao et al., 2002; Hord et al., 2006), which suggests that cell–cell signaling events are critical for tapetum and pollen development (Feng and Dickinson, 2010b). We found 26 receptor kinases down-regulated in *dyt1* mutant, 3 of them were *DYT1*-specific genes, 2 of them were *TDF1*-specific genes, 21 of them were common genes (Supplementary Table S17). We also found RALF type peptides were common genes (**Supplementary Figure S2**). These suggest that tapetum cell keeps the cell-cell communication during different development stage. Genes functioned in transport were differentially and commonly expressed in these mutants (Supplementary Table S18), indicating these factors controls a highly ordered transport system in tapetum for pollen development. Therefore, tapetum cells evolve a complicated gene regulatory network for its ordered development and finally PCD while efficiently communicates with other plant cells for pollen formation.

## Accession Number

Microarray data from this article can be found in Gene Expression Omnibus (GEO) under the accession number GSE102528. The RNA-Seq data for tapetal cells of *Arabidopsis* stages 6–7 anthers and stages 8–10 anthers have been deposited in Sequence Read Archive (SRA) repository under the accession number SRS2426392 and SRS2426393.

## Author Contributions

The experiments were conceived and designed by: Z-NY. The experiments were performed by: D-DL, JZ. The data were analyzed by: D-DL, J-SX, and JZ. The paper was written by: J-SX, D-DL, and Z-NY.

## Conflict of Interest Statement

The authors declare that the research was conducted in the absence of any commercial or financial relationships that could be construed as a potential conflict of interest.

## Footnotes

1 http://www.illumina.com/

2 http://www.arabidopsis.org

3 http://www.megasoftware.net/

4 https://www.r-project.org/

## Supplementary Material

FIGURE S1 Heat map of mRNA expression profiles of ralf-like genes of *MS188* in *dyt1, tdf1, ams*, and *ms188* mutants.

FIGURE S2 Heat map of mRNA expression profiles of peroxidases of *TDF1* in *dyt1, tdf1, ams* and *ms188* mutants.
