# Supplementary figures and images for: Gene Regulatory Network for Tapetum Development in Arabidopsis thaliana
Source: Front Plant Sci. 2017 Sep 12;8:1559. doi: 10.3389/fpls.2017.01559 (PMC5601042; doi:10.3389/fpls.2017.01559)

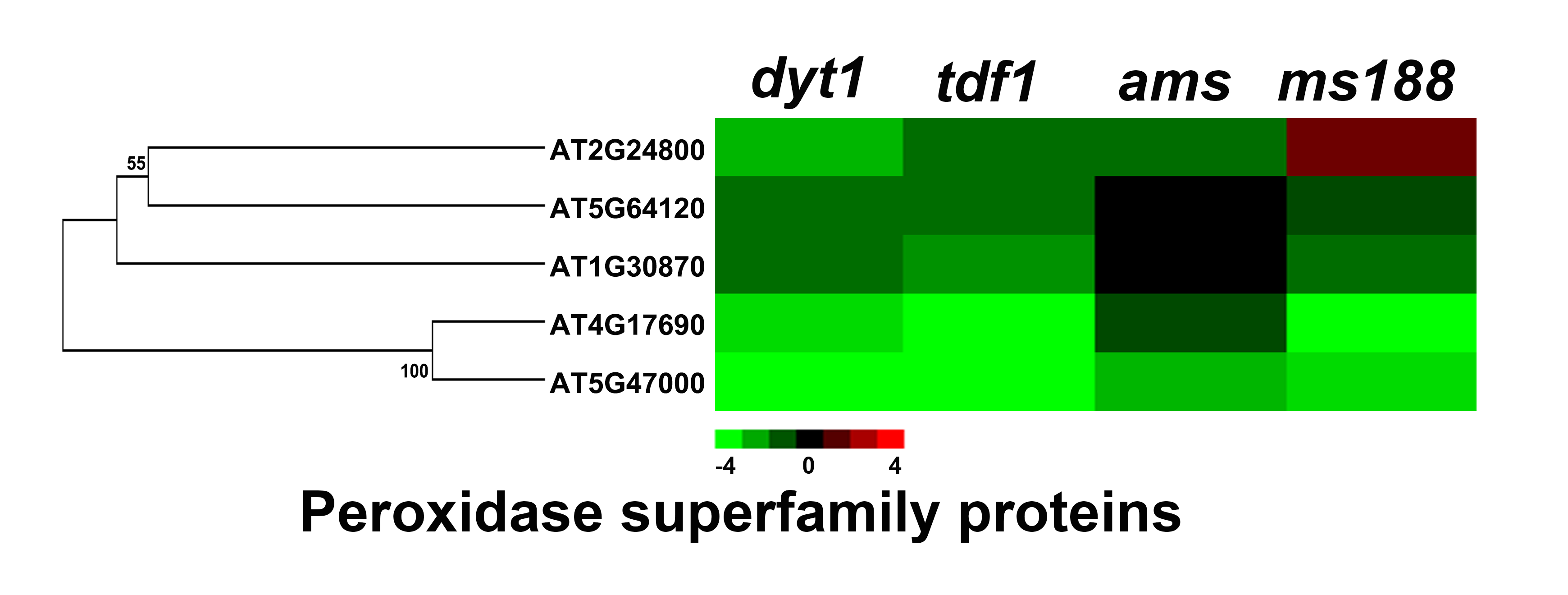

Supplement: FIGURE S1 — Heat map of mRNA expression profiles of ralf-like genes of MS188 in dyt1, tdf1, ams, and ms188 mutants. [file Image_1.TIF]

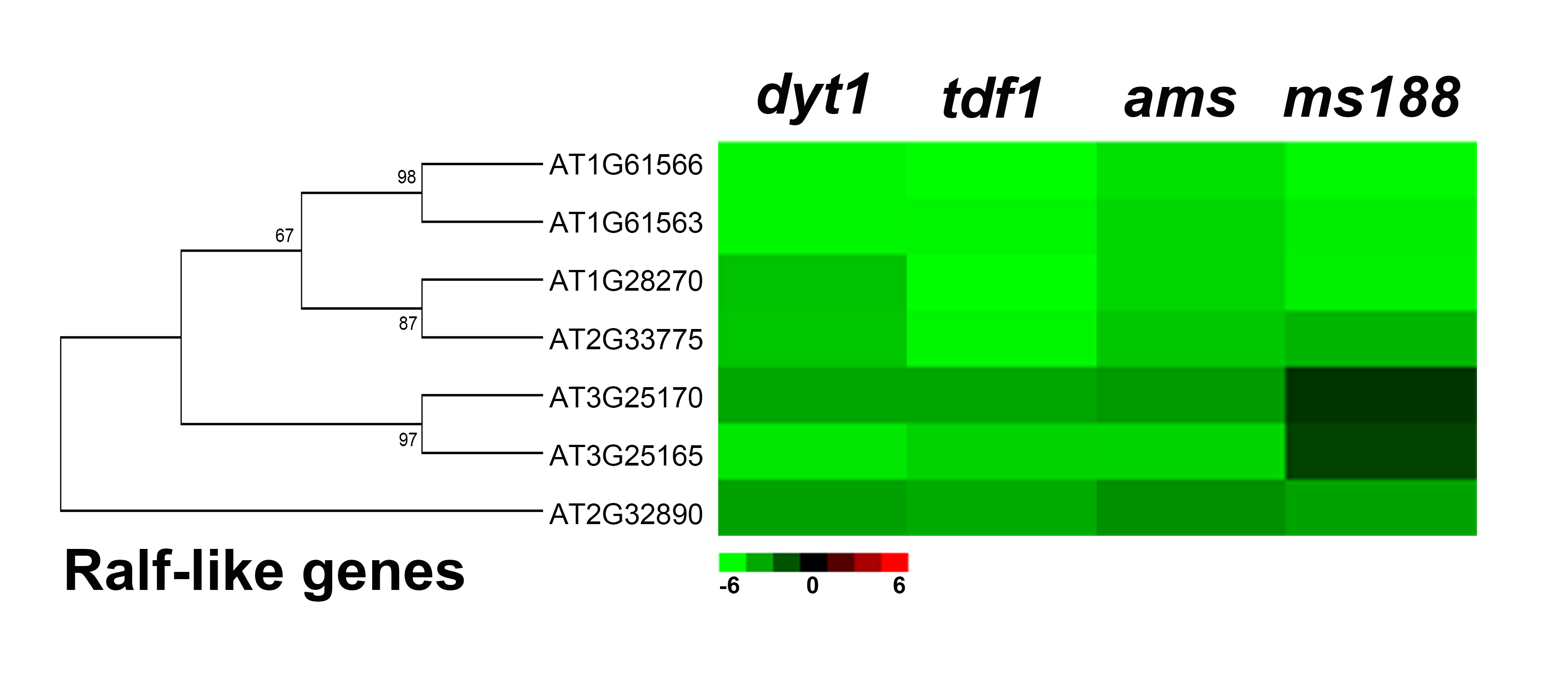

Supplement: FIGURE S2 — Heat map of mRNA expression profiles of peroxidases of TDF1 in dyt1, tdf1, ams and ms188 mutants. [file Image_2.TIF]
